# *Ent*-Kaurane Diterpenoids from *Coffea* Genus: An Update of Chemical Diversity and Biological Aspects

**DOI:** 10.3390/molecules30010059

**Published:** 2024-12-27

**Authors:** Víctor de C. Martins, Maria Alice E. da Silva, Valdir F. da Veiga, Henrique M. G. Pereira, Claudia M. de Rezende

**Affiliations:** 1 Aroma Analysis Laboratory (LAROMA), Institute of Chemistry, Federal University of Rio de Janeiro, Rio de Janeiro 21941-909, Brazil; m.aliceesteves@ufrj.br (M.A.E.d.S.); crezende@iq.ufrj.br (C.M.d.R.); 2 Brazilian Doping Control Laboratory (LBCD), Institute of Chemistry, Federal University of Rio de Janeiro, Rio de Janeiro 21941-909, Brazil; henriquemarcelo@iq.ufrj.br; 3 Chemistry Section, Military Institute of Engineering, Rio de Janeiro 22290-270, Brazil; valdir.veiga@ime.eb.br

**Keywords:** coffea, green and roasted coffee, *ent*-kaurane, new compounds, antidiabetic potential

## Abstract

Coffee is one of the most important beverages in the world and is produced from *Coffea* spp. beans. Diterpenes with *ent*-kaurane backbones have been described in this genus, and substances such as cafestol and kahweol have been widely investigated, along with their derivatives and biological properties. Other coffee *ent*-kaurane diterpenoids have been reported with new perspectives on their biological activities. The aim of this review is to update the chemical diversity of *ent*-kaurane diterpenoids in green and roasted coffee, detailing each new compound and reporting its biological potential. A systematic review was performed using the bibliographic databases (SciFinder, Web of Science, ScienceDirect) and specific keywords such as “coffea diterpenes”, “coffee diterpenes”, “coffee ent-kaurane diterpenes” and “coffee diterpenoids”. Only articles related to the isolation of coffee *ent*-kaurane compounds were considered. A total of 146 compounds were related to *Coffea* spp. since the first report in 1932. Different chemical skeletons were observed, and these compounds were grouped as furan-type, oxidation-type, rearrangement-type, lacton-type, and lactam-type, among others. In general, the new coffee diterpenoids showed potential as antidiabetic, antidiapogenic, α-glucosidade inhibition, antiplatelet activity, and Cav.3 inhibitors agents, revealing the possibilities for the design, discovery, and development of new drugs.

## 1. Introduction

The *Coffea* L. genus (Rubiaceae family) consists of an important Angiosperms taxon with around 132 species. In general, this genus is characterized by small trees or shrubs (4–8 m) and the production of small red drupe fruits (cherries) [1]. Through different postharvest methodologies, coffee beans are obtained and are one of the most appreciated beverages worldwide. *Coffea* spp. were originally found in African, tropical Asian, and Oceanian regions [1]. The most known history indicates that coffee was discovered in the 10th century by an Ethiopian shepherd when observing the unusual behavior of animals that consumed the fruits present in the region. The stimulant effect and sensorial aspects were responsible for its high valorization in subsequent centuries, mainly in Europe and North America, contributing to the introduction of coffee plantations in other regions, such as Southern America and temperate Asia [2,3].

Currently, there is an extensive coffee industry that can meet the high demand for this commodity. In 2023, world production reached 178.0 million 60-kg coffee bags, with an estimate consumption of 177.0 million 60-kg coffee bags. The main producer countries are Brazil, Vietnam, Colombia, Indonesia, and India. Brazil is the leader in general coffee bean exportation, mainly to the USA and the European Union [4,5]. Among different coffee species, two commercial species are highly consumed: *C. arabica* L. (Arabica coffee) and *C. canephora* Pierre ex A.Froehner (Robusta coffee) [6,7]. Brazil is the world’s leading producer of Arabica coffee beans, a higher economic value species, while Vietnam is the largest producer of Robusta coffee in the world [4].

The chemical composition of coffee beans remains the principal target for the investigation of different aspects, such as coffee quality, occurrence of frauds, and valorization of special coffee [5]. Green coffee mainly contains carbohydrates, soluble proteins, and lipid compounds. For example, the lipid fraction of Arabica coffee beans can reach 17% (*w*/*w* dry weight). There is an important subclass of specialized plant metabolites, known as *ent*-kaurane diterpenoids, in which coffee beans are the main source of the compounds cafestol and kahweol (up to 18.5%, as fatty acid derivatives) [7,8]. Another known *ent*-kaurane diterpenoid, 16-*O*-methylcafestol, has been considered present exclusively in Robusta coffee for a long time and has been used as an authenticity marker for commercial coffee blends [5,9]. In recent years, advanced analytical techniques have also allowed the detection of 16-*O*-methylated diterpenoids in *C. arabica* [10,11]. In addition to this differentiation between botanical species, the roasting process of beans leads to the formation of oxidation products; some compounds are widely known as dehydrocafestol and dehydrokahweol and are transferred to the beverage [8,12].

The matrix complexity of coffee indicates the presence of several other compounds with similar chemical skeletons. In the scientific literature, some comprehensive reviews have been published in recent decades to report all the diterpenoids found in *Coffea* spp. and their biological activities, such as anticancer and anti-inflammatory potential [6,7,12]. However, there are many recent reports of other coffee *ent*-kaurane diterpenoids, mainly in the post-pandemic years (after the year 2020). New perspectives on the biological activities of these compounds have also been related since the last decade. The aim of this review is to update the chemical diversity of these compounds, detailing each new compound isolated through the coffee matrix, and reporting the biological potential of these compounds.

To this end, a systematic review was performed using the main bibliographic databases (SciFinder, Web of Science, and ScienceDirect). Specific keywords were employed, such as “coffea diterpenes”, “coffee diterpenes”, “coffee ent-kaurane diterpenes,” and “coffee diterpenoids”. The research was performed between June and August 2024, resulting in around one hundred scientific articles. Scientific articles that did not involve the isolation of *ent*-kaurane compounds from green and roasted coffee matrices were not considered in this review. The molecular structures of all related compounds were described using ChemDraw^®^ software.

## 2. Biosynthesis of *Ent*-Kaurane Diterpenoids

Coffee diterpenoids belong to the isoprenoid group, a major class of natural products, with more than 100,000 compounds described in the scientific literature [13]. These compounds have high chemical structure diversity and numerous functional activities. One of the most important isoprenoid classes is terpenoid compounds, polymers from C_5_ basic units (isoprene units) with different sizes through different coupling reactions, such as classical chain elongation. In general, the term “terpenoids” refers to natural hydrocarbons that contain heteroatoms, such as O and N, in their functional groups, whereas “terpenes” only consist of hydrocarbons. Furthermore, a large range of terpenoids (e.g., monoterpenes, sesquiterpenes, diterpenoids, triterpenoids, steroids, and carotenoids) have vital importance in several plant functions, as well as responses against biotic and abiotic stress factors, pollinator attractions, and protective barriers against pathogens and predators, among others. These large functions led to the classification of isoprenoids mainly as specialized plant metabolites [14,15,16].

**Figure 1:**
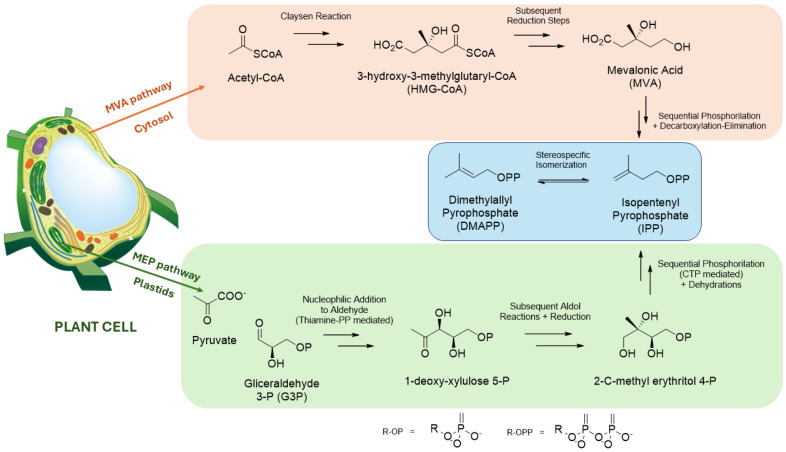
General representation of the biosynthetic route for formation of the isoprene units (C_5_), the precursor of isoprenoids.

*Ent*-kaurane diterpenoids are biosynthesized via three complex processes involving several enzymatic reactions. In the first plant process, C_5_ units, dimethylallyl diphosphate (DMAPP), and isopentenyl diphosphate (IPP), which are precursors of general terpenoids, are produced. In recent decades, two different mechanisms have been described for the biosynthesis of these compounds: the mevalonate pathway (MVA) and methylerythritol phosphate (MEP). These mechanisms differ in substrate and plant cell distribution. The MVA pathway occurs mainly in the cytosol, and the IPP unit is formed from the initial condensation of acetyl-coenzyme A (acetyl-CoA) and the subsequent reduction of mevalonate. In addition, in chloroplasts, IPP is formed from pyruvate and glyceraldehyde 3-phosphate (G3P), glycolysis compounds of the MEP pathway [14,15]. Figure 1 shows both pathways for the production of IPP and DMAPP. The mechanism of MEP pathways has mainly been described for the formation of general diterpenoids [14,17].

**Figure 2:**
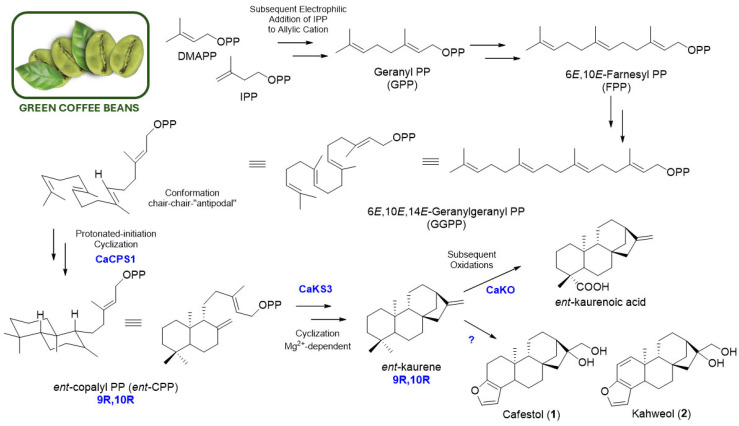
Current biosynthetic mechanism of ent-kaurane skeleton for diterpenoids found in coffee beans, adapted from Peters (2010) and Ivamoto-Suzuki et al. (2023) [17,20].

The second plant process initially consists of the classical 1–4’ condensation of IPP and DMAPP (C_5_), in “head-to-tail” mechanism intermediated by prenyltransferases, resulting in the production of the *E*,*E*,*E*-geranylgeranyl pyrophosphate (GGPP, C_20_) [14,18,19]. This unit is a common substrate for the formation of all diterpenoids, described as plant and microbial metabolites. After this formation, the last mechanism involves cyclization reactions, which are the most complex process. It led basically to the formation of two perhydronaphtalene bicyclic intermediates, resulting in enantiomeric products with distinct optical configurations at carbons C-5, C-9, and C-10. These aspects provide a different subclass of diterpenoids, such as labdanes, clerodanes, abietanes, kauranes, and taxanes. The stereochemistry of the intermediate depends on the prochiral conformation of GGPP, in which the formation of *ent*-kaurane skeletons only occurs through the chair-chair-“antipodal” conformation [19,20]. The action of class I and/or II cyclases also plays an important role in this metabolism. For example, in the soil bacterium *Bradyrhizobium japonicum*, the biosynthesis of *ent*-kaur-16-ene was described through class II protonation-mediated cyclization of GGPP for the formation of *ent*-copalyl diphosphate (*ent*-CPP) and the subsequent class I cyclization by metal-dependent ionization, catalyzed by *ent*-copalyl diphosphate synthase (*ent*-CPPS) [18]. Recently, among many contributions to diterpene biosynthesis [21,22,23], Ivamoto-Suzuki et al. [17] performed genomic and transcriptomic investigations of *Coffea arabica* beans. This research indicated that, in coffee beans, CACPS1 catalyzes the cyclization reaction of GGPP to *ent*-CPP, and the CAKS3 protein facilitates the final cyclization to the *ent*-kaurane skeleton. All the mentioned information is summarized in Figure 2. This research will lead to future studies on the identification of other enzymes involved in the formation of other coffee *ent*-kaurane diterpenoids, such as cafestol (**1**) and kahweol (**2**).

## 3. An Overview of Diterpenoids Derived from Cafestol and Kahweol

The main coffee *ent*-kaurane diterpenoids are cafestol (C, **1**) and kahweol (K, **2**). These compounds have a specific *ent*-kaurane skeleton with a furan ring fused to an A ring through methyl migration (C-18 or C-19) and final cyclization [12,24]. The first reports described C&K diterpenoids as exclusive compounds from coffee species. However, cafestol has also been found in *Tricalysia dubia*, another species of the Rubiaceae family [7,25]. Currently, coffee is the main source of these diterpenoids, which are distributed in almost all plant tissues (except leaves). Higher C&K concentrations were related to the flower buds and mature fruits of *C. arabica* L., reaching levels up to 2.2% (dry-weight beans) [12,22].

These diterpenoids have been investigated since the 30th decade of the last century. The first report was published in 1932 by American scientists R. Bengis and R. Anderson. They described the crystalline compound isolated from the unsaponifiable fraction of coffee bean oil and named it kahweol, according to the Arabic word *qahweh* (coffee). Using available tools, these authors related the melting point, optical rotatory dispersion, and chemical instability to atmospheric oxygen, light, heat, and mineral acids [26].

In the following years, another compound that is more stable than kahweol was discovered in coffee oil. Firstly, in 1938, the German scientists Karl Slotta and Klaus Neisser described this compound and named it cafesterol [27,28]. Several scientific articles were published until the 60’s decade for the determination of the chemical structure of this diterpenoid [29,30,31]. The main studies developed by Professor Carl Djerassi revealed complete information on this chemical structure through the experiments of chemical derivatization, ultraviolet absorption, and infrared spectroscopy. Cafestol, as renamed by Djerassi, has a furanoid phyllocladene skeleton, a tetracylic system (A-D) formed by 6/6/6/5 C atoms with bicyclo[3.2.1]octane for the C and D rings and a furan ring fused to the A ring [32,33,34,35,36]. In addition to this contribution, the chemical structure of kahweol was finally determined by Hans Kaufmann and Achintya Sen Gupta, also as a furan-type *ent*-kaurane diterpenoid [37,38,39,40].

**Figure 3:**
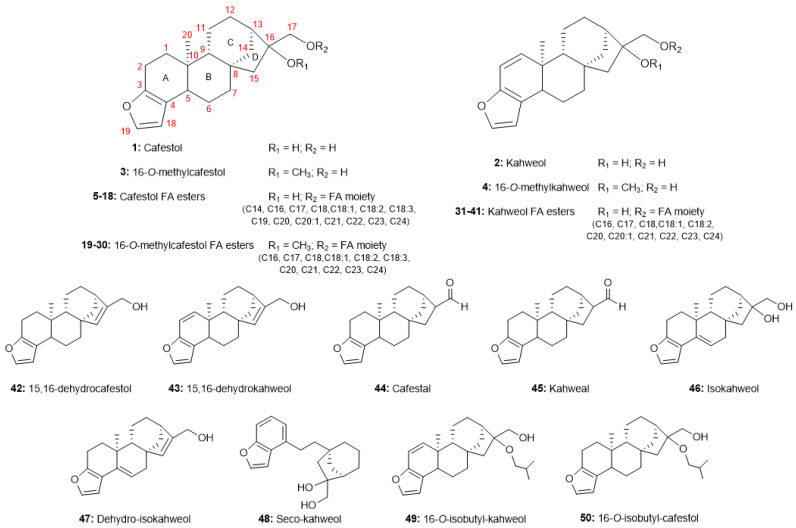
Main C&K derivatives found in commercial coffee species.

Other compounds with similar skeletons have been described for *Coffea* spp. beans (Figure 3). 16-*O*-methylcafestol (16-OMC, **3**) was identified by Pettit et al. in 1987 and thus confirmed by Speer & Mischnick in 1989 as an *ent*-kaurane diterpenoid with restricted occurrence in *C. canephora* (Robusta) beans at that time [9,41]. Both studies mainly employed the technique of gas chromatography coupled with mass spectrometry (GC-MS) for the chemical structure characterization plus derivatization reactions, fragmentation pattern studies, and proton nuclear magnetic resonance (^1^H NMR). In addition, in the same year, Speer detailed a procedure for the separation of these diterpenoids (C, K, 16-OMC) in coffee samples by adsorption chromatography on a silica column for characterization through GC-MS and high-performance liquid chromatography (HPLC) [42]. This evaluation was initially important for distinguishing between botanical species. For example, through GC-MS analysis, de Roos et al., in 1997, investigated the Wild African *Coffea* beans and revealed that 16-OMC was only found in *C. canephora*, *C. stenophylla* and *C. liberica* var. *dewevrei* [43]. Thus, 16-OMC has been considered a chemical marker for commercial Robusta coffee because of its absence in commercial Arabica coffee and its higher thermal stability. In Germany, a specific norm called DIN 10779 (1999) was developed, and the quantification of 16-OMC is recommended for the evaluation of coffee blends [12]. Different quantification methodologies, including NMR experiments (mainly through the proton signal of the methoxy group at 3.16 ppm for 16-OMC), have been developed in the last decades for this evaluation [10,11,44,45,46,47]. Another diterpenoid was tentatively identified by de Roos et al. in *C. stenophylla* as 16-*O*-methylkahweol (16-OMK, **4**) through the comparison of the 16-OMC fragmentation pathway after the silylation reactions [43]. 16-OMK was also initially found in Robusta coffee beans [12]. Currently, 16-O-methylated diterpenoids have already been detected in green and roasted *C. arabica* beans through advanced analytical techniques such as high-resolution NMR (600 MHz) and ultra-high performance liquid chromatography-tandem mass spectrometry (UHPLC-MS/MS) [10,11].

In the lipid fraction of coffee beans, these furan-type *ent*-kaurane diterpenoids can be found in the free form as di-alcohols and mainly in the conjugated form with several fatty acids (FA) as C-17 esters, reaching levels of up to 0.4% and 18.5%, respectively [8,12,48]. In 1962, Hans Kaufmann and collaborator R. Hamsagar first described the occurrence of cafestol esters linked to FA through the thin-layer chromatography (TLC) analysis [37]. In the following years, the identification of fatty acid ester diterpenoids in *C. arabica* and *C. canephora* beans increased, mainly through the application of gel permeation chromatography, HPLC-UV in reverse-phase, and/or GC-MS analysis. Thirty seven (**5**–**41**) compounds were described in the literature such as fourteen cafestol FA esters (C14, C16, C17, C18, C18:1, C18:2, C18:3, C19, C20, C20:1, C21, C22, C23, C24), eleven kahweol FA esters (C16, C17, C18, C18:1, C18:2, C20, C20:1, C21, C22, C23, C24) and twelve 16-OMC FA esters (C16, C17, C18, C18:1, C18:2, C18:3, C20, C20:1, C21, C22, C23, C24) [41,48,49]. In 1982, Lam et al. isolated for the first time cafestol palmitate (C16, **6**) and kahweol palmitate (C16, **31**) through chromatographic separation (including silver nitrate-impregnated thin-layer chromatography) from the petroleum ether extract of green coffee beans. To confirm their identification, the pure compounds, alcohol analogs, and acetylated derivatives were fully characterized by classical spectroscopic methods, such as infrared analysis (IR), high-resolution mass spectrometry (HRMS), and NMR spectroscopy [50]. In addition, the FA ester diterpenoids found in higher amounts in coffee beans are usually linked to palmitic (C16), linoleic (C18:2), oleic (18:1), stearic (C18), arachidic (C20), and behenic acid (C22) [7,12,51].

In addition, the chemistry of coffee-roasted beans has also been investigated in the scientific literature, mainly through the evaluation of the diterpenoid profile. The levels of these compounds in coffee-roasted beans depend on the different factors in which the roasting process impacts the chemical composition of the beans [7,8,12]. The thermal methods led to the formation of several known oxidation products such as 15,16-dehydrocafestol (**42**), 15,16-dehydrokahweol (**43**), cafestol (**44**), kahweal (**45**), isokahweol (**46**) and dehydro-isokahweol (**47**) [12]. In 2009, Scharnhop and Winterhalter developed a high-speed countercurrent chromatography (HSCCC) method for the isolation of the natural diterpenoids of *C. arabica* and *C. canephora* var. *robusta*, including the roasting products 15-16-dehydrocafestol and 15,16-dehydrokahweol. This methodology, in addition to preparative HPLC, provides isolated compounds with a minimum of 87% purity [52]. Recently, Novaes et al. investigated the influence of different degrees of roasting on the diterpenoid composition of green Arabica coffee beans. Sixteen compounds were tentatively identified by GC-MS as ten derived-kahweol (seven dehydro-kahweol isomers, kahweal—**45**, seco-kahweol—**48**, 16-*O*-isobutyl-kahweol—**49**) and six derived-cafestol (three cafestol derivatives isomers, 15,16-dehydro-cafestol—**42**, cafestal—**44**, and 16-*O*-isobutyl-cafestol—**50**) [8]. This information could lead to new research on the thermal products of cafestol and kahweol, including extensive identification by spectroscopy analysis for structural elucidation.

## 4. Other Classical *Ent*-Kaurane Diterpenoids from *Coffea* Genus

Due to the high potential, the investigation of the diterpenoids from the *Coffea* genus was expanded to evaluate different aspects (e.g., botanical species, distribution in the plant organs, roasting process, and chemical diversity). This trend started in the 70’s decade in which three furan-type *ent*-kaurane diterpenoids were initially isolated and fully characterized for different *Coffea* spp.

Mascaroside (**51**) was the first glucoside diterpenoid found in coffee beans isolated from *C. vianneyi* beans, a Malagasian species. In addition to the glucose unit at C-17 and two hydroxyl groups at C-11 and C-15 in the diterpenoid skeleton, this compound presents a keto group at C-2, a common oxidation site observed for other diterpenoids that were later isolated [53,54]. Another glucoside compound, 11-*O*-(*β*-*O*-glucopyranosy1)-cafestol-2-one (**52**), named mozambioside, was isolated from *C. arabica* and *C. pseudozanguebariae* beans and characterized by X-ray crystallography, MS, and NMR spectroscopy [55]. In 1994, Hasan et al. developed an unusual scientific research with *C. bengalensis* leaves, a Bangladeshi species, and described, through a structure elucidation study, a C-11,C-16-epoxide diterpenoid, named bengalensol (*ent*-18(4→19)-*abeo*-3,18:11α,16α-diepoxy-17-hydroxy-3,18-kauradien-2-one, **53**) [56].

In the same period, another class of diterpenoids found in Arabica coffee beans was largely known as oxidation-type *ent*-kaurane diterpenoids (mainly with oxygenated groups at C-19). The first isolated compound was *ent*-16-kauren-19-ol (**54**), fully characterized by Wahlberg and Enzell through the isolation from *C. arabica* beans [57]. Several studies by the German scientist Gerhard Spiteller led to the discovery of different glucoside oxidation-type *ent*-kaurane diterpenoids [58,59,60,61,62]. Furthermore, these investigations confirmed the presence of atractylosides in *Coffea* spp., a toxic glucoside diterpenoid subclass found initially in the rhizomes of *Atractylis gummifera*, but largely distributed in plants [63,64].

Initially, in 1974, Ludwig et al. isolated atractyligenin from the aqueous extract of roasted beans (**55**), an aglycone of the main atractylosides found in coffee [58]. From green *C. arabica* beans, other atractylosides such as 2-*O*-*β*-*D*-glucopyranosyl-atractyligenin (**56**), 2-*O*-(3′-*O*-*β*-*D*-glucopyranosyl-2′-*O*-isovaleryl-*β*-*D*-glucopyranosyl)-atractyligenin (**57**), and 2-*O*-(2′-*O*-isovaleryl-β-*D*-glucopyranosyl)-atractyligenin (**58**) were also isolated In the following scientific researches [59,61]. These diterpenoids have a similar chemical structure to glycoside molecules linked to C-2 (plus isovaleric ester-linked to glycoside residue at C-2′ in some compounds) and free carboxylic and hydroxyl groups (C-4 and C-15, respectively). Another glucoside oxidation-type diterpenoid isolated by the same research group was 19-*O*-*β*-*D*-glucopyranosyl-9*β*,16*α*,17-trihydroxykauran-18-ate, named cofarylosid (**59**), from green *C. arabica* beans [60]. In general, these discoveries contributed to the identification of new diterpenoids in *Coffea* spp. and their biological activities in the following decades.

## 5. Recent Discoveries of Coffee Diterpenoids

**Figure 4:**
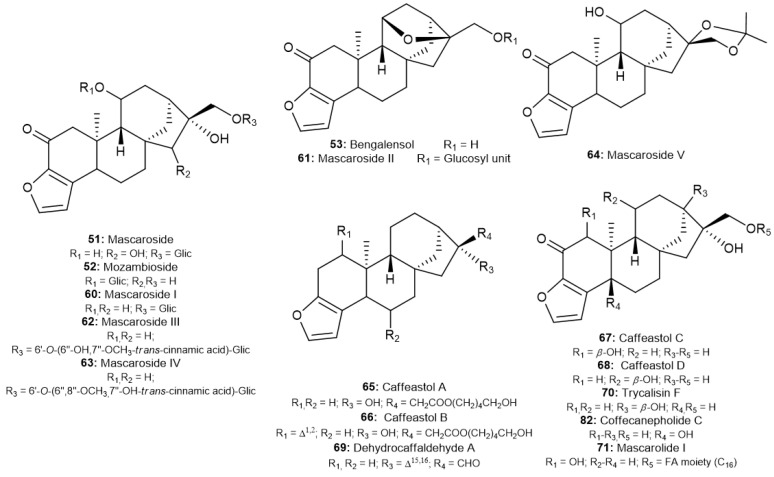
Furan-type *ent*-kaurane diterpenoids found in *Coffea* spp.

**Figure 5:**
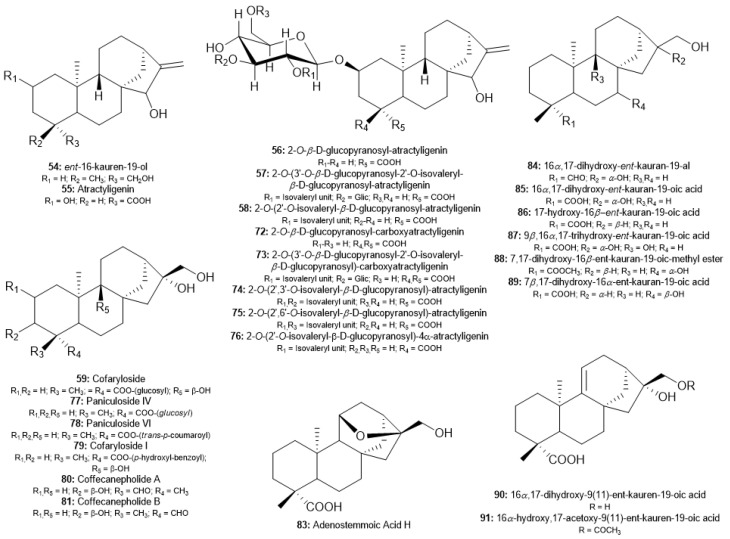
Chemical diversity of oxidation-type *ent*-kaurane diterpenoids, mainly atractylosides, found in *Coffea* spp.

**Figure 6:**
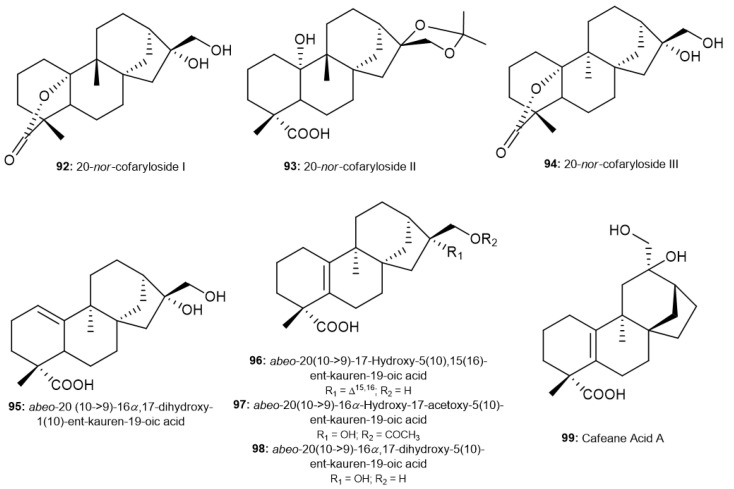
Rearrangement-type *ent*-kaurane diterpenoids found in *Coffea* spp.

**Figure 7:**
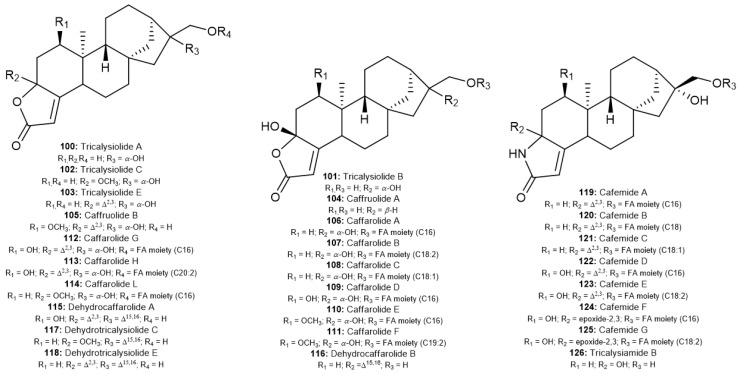
Lactone-type and lactam-type *ent*-kaurane diterpenoids found in *Coffea* spp.

**Figure 8:**
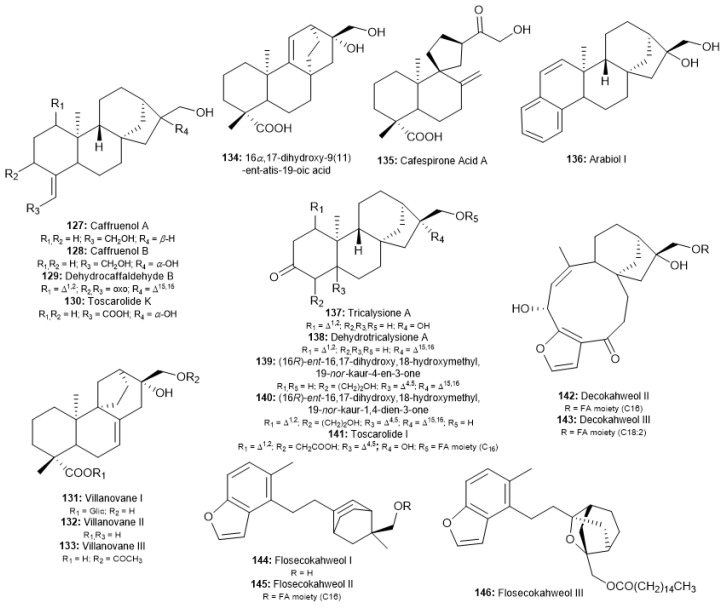
Chemical diversity of coffee *ent*-kaurane diterpenoids with different skeletons.

Several phytochemical evaluations of *Coffea* spp. have been published over the last decade. Most of these scientific articles involved coffee beans cultivated in Yunnan province, located in the People’s Republic of China, led by Chinese Professor Minghua Qiu. A common characteristic is the extraction of diterpenoids from higher amounts of material (greater than 10 kg) and sequential chromatographic separations. This methodology provides a small quantity of pure compounds (mainly 1.5–20 mg) due to the fact that these diterpenoids are minor constituents of coffee extracts. However, extensive structural elucidation, including one- and two-dimensional NMR spectroscopy, has been developed for each compound. In general, these studies led to the identification of another eighty-seven coffee *ent*-kaurane diterpenoids. In this review, these compounds were divided into five main groups, according to the large diversity: “furan-type”, “oxidation-type”, “rearrangement-type”, “lactone-type/lactam-type” and “other-type”. Table 1 summarizes all available information on these phytochemical studies, and Figure 4, Figure 5, Figure 6, Figure 7 and Figure 8 describe the chemical diversity of these compounds.

### 5.1. Furan-Type Ent-Kaurane Diterpenoids

Other furan-type diterpenoids from *Coffea* spp. have been described in recent years (Figure 4). A series of mascaroside I-V (**60**–**64**), similar to the mascaroside skeleton described by Ducruix et al., were isolated by Shu et al. and Chu et al. from roasted and green Arabica coffee from Yunnan [53,54,66,67]. Other C&K derivatives have also been described in these studies. Hu et al. isolated from the roasted coffee of the same location two rare C&K derivatives with 6-hydroxyhexanoyl at C-17, named caffeastol A and B (**65**–**66**), and an unknown degradation product of cafestol, with C15-C16 double bond and aldehyde group at C-17, named as dehydrocaffaldehyde A (**69**) [71].

The study of different matrixes led to the identification of other furan-type diterpenoids. Al-Romaima et al. described the compounds caffeastol C and D (**67**–**68**) and trycalisin F (**70**), with hydroxyl groups at different positions in the carbonic skeleton, from cherries pulp of Arabica coffee [76]. Similarly, Hoang et al. isolated, from *C. canephora* trunks, the coffecanepholide C (**82**), with an unusual hydroxyl group at C-5 [77]. Another furane-type compound, mascarolide I (**71**), was described by Wang et al. from the same roasted Yunnan coffee [75].

### 5.2. Oxidation-Type Ent-Kaurane Diterpenoids

Among the many coffee diterpenoid skeletons with oxidation degrees at C-18 and/or C-19 (Figure 5), the subclass of atractylosides remains the main *ent*-kaurane diterpenoids found in different coffee matrices. Atractyligenin (**55**), 2-*O*-*β*-*D*-glucopyranosyl-atractyligenin (**56**), 2-*O*-(3′-*O*-*β*-*D*-glucopyranosyl-2′-*O*-isovaleryl-*β*-*D*-glucopyranosyl)-atractyligenin (**57**), and 2-*O*-(2′-*O*-isovaleryl-*β*-*D*-glucopyranosyl)-atractyligenin (**58**) were also isolated in these studies published since the last decade [65,66,67,69,77]. However, the other five atractylosides were first described in *Coffea* spp. Lang et al. isolated two carboxyatractylosides, 2-*O*-*β*-D-glucopyranosyl-carboxyatractyligenin (**72**) and 2-*O*-*β*-D-(3′-*O*-*β*-D-glucopyranosyl-2′-*O*-isovaleryl)-glucopyranosyl-carboxyatractyligenin (**73**), from green commercial coffee for the first time, and proposed their identification through extensive NMR spectroscopy and mass spectrometry studies [65]. Hu et al. described the isolation from roasted Yunnan coffee of two different atractyloside compounds with two isovaleryl units linked to pyranosyl ring of glucose in different positions and named as 2-*O*-(2′,3′-*O*-isovaleryl-*β*-*D*-glucopyranosyl)-atractyligenin (**74**) and 2-*O*-(2′,6′-*O*-isovaleryl-*β*-*D*-glucopyranosyl)-atractyligenin (**75**) [71]. Finally, a new compound with an *α*-carboxyl group linked to C-4, 2-*O*-(2′,3′-*O*-isovaleryl-*β*-*D*-glucopyranosyl)-4-*α*-atractyligenin (**76**) was obtained by Shu et al. and Chu et al. from extracts of green and roasted Yunnan coffee [66,67].

In roasted Yunnan coffee, other new oxidation-type diterpenoids were related by these authors, such as paniculoside VI (**78**), cofaryloside I (**79**), adenostemmoic acid H (**83**), 16*α*,17-dihydroxy-9(11)-ent-kauren-19-oic acid (**90**), and 16*α*-hydroxy-17-acetoxy-9(11)-ent-kauran-19-oic acid (**91**) [66,72]. In addition, in research on *C. canephora* trunks from Vietnam, Nguyen et al. isolated two new *ent*-kaurane diastereomers, named coffecanepholide A and B (**80**–**81**), through the different positions of the aldehyde group at C-18 or C-19 [74]. In the last decade, other known *ent*-kaurane diterpenoids have also been isolated from *Coffea* spp., including paniculoside IV (**77**) and a series of *ent*-kauran-19-oic acid derivatives (**84**–**89**) [66,69,76].

### 5.3. Rearrangement-Type Ent-Kaurane Diterpenoids

Eight new *ent*-kaurane diterpenoids found in *Coffea* spp. showed migration of the methyl group C-20 of C-10 to C-9 (Figure 6). In 2014, Shu et al. isolated the first compounds, 20-*nor*-cofaryloside I and II (**92**–**93**), from roasted Yunnan coffee. In addition to the rearrangement of C-20 methyl, these compounds have a specific structure, in which compound **92** has a lactone ring between the hydroxyl group at C-10 and C-19 carboxyl group, and compound **93** has a spiro carbon between hydroxyl groups at C-16 and C-17 [66]. 20-*nor*-Cofaryloside III (**94**) was isolated by Hong et al. in a specific cultivar (S288) of Arabica coffee, with a different configuration of C-20 in comparison to 20-*nor*-cofaryloside I [72]. Five other compounds with different unsaturation bonds in the *ent*-kaurane skeleton (∆^1,10^, ∆^5,10^, and/or ∆^15,16^) were also isolated from roasted Yunnan coffee, such as the *abeo*-C-20 compounds *abeo*-20-(10→9)-16*α*,17-dihydroxy-1(10)-ent-kauren-19-oic acid (**95**), *abeo*-20(10→9)-17-hydroxy-5(10),15(16)-ent-kauren-19-oic acid (**96**), *abeo*-20(10→9)-16*α*-hydroxy-17-acetoxy-5(10)-ent-kauren-19-oic acid (**97**), *abeo*-20(10→9)-16*α*, 17-dihydroxy-5(10)-ent-kauren-19-oic acid (**98**), and cafeane acid A (**99**) [71,72,73].

### 5.4. Lactone-Type/Lactam-Type Ent-Kaurane Diterpenoids

The first lactone-type *ent*-kaurane diterpenoids found in *Coffea arabica* were described in two studies by Wang et al. [68,69]. From green coffee beans, ten novel lactone compounds, caffruolide A and B and caffarolide A–G (**104**–**113**), were isolated as known tricalysiolide A, B, C, and E (**100**–**103**). In addition, possible oxidation products from roasted Yunnan coffee beans were first isolated by other authors, such as dehydrocaffarolide A, dehydrocaffarolide B, and dehydrotricalysiolide C and E (**115**–**118**) [71,75,76]. Recently, a novel lactone compound, named caffarolide L (**114**), was described by Wang et al. [75].

Other diterpenoids with specific skeletons were observed in roasted coffee beans. In these compounds, the lactam ring is conjugated to the A ring instead of the furan and lactone rings. The main study of lactam-type diterpenoids by Hu et al. isolated seven new coffee diterpenoids, named cafemide A–G (**119**–**125**), with FA residues (C16, C18, C18:1, or C18:2) linked to C-17 [70]. Another lactam-type, tricalysiamide B (**126**), was also isolated from Arabica coffee pulp by Al-Romaima et al. [76]. The chemical structures of these compounds are shown in Figure 7.

### 5.5. Other-Type Ent-Kaurane Diterpenoids

In recent literature, unusual skeletons of coffee diterpenoids have been reported as a few compounds (Figure 8). It was observed a specific structural characteristic for each compound, e.g., the occurrence of C-4/C18 double bond (∆^4,18^), the cleavage of C-5/C-10 or C-9/C-10 to give *seco*-compounds, an aromatic ring fused to A ring, the rearrangement of C ring bonds and formation of *abeo*-14-(13→12), similar to “atisane” skeleton, or *abeo*-14-(8→9,13→12), similar to “Villanova” skeleton, among others. Due to the few unusual *ent*-kaurane diterpenoids of each subclass, all compounds are grouped in this review as “other-type”.

Four compounds were described for the first time and showed loss of the C-19 methyl group and the presence of double bonds as ∆^4,18^. Wang et al. isolated caffruenol A and B (127–128) from green Yunnan coffee [69]. Al-Romaima et al. reported the isolation of toscarolide K (**130**), in addition to caffruenol B, from the cherries pulp of *C. arabica* [76]. The classical double bond at C-15/C-16 caused by the dehydration reaction in the roasting process of coffee was observed in the skeleton of another compound named dehydrocaffaldehyde B (**129**). This compound was isolated by Hu et al. from roasted Yunnan coffee [71].

The rearrangement of different bonds in the C-D rings provides a specific skeleton for coffee diterpenoids. Initially, five new *ent*-kaurane diterpenoids were isolated exclusively from roasted Yunnan coffee. The study of Shu et al. was the first to isolate a coffee diterpenoid with “villanova” skeleton, named villanovane I (**131**) [66]. Hu et al. and Hong et al. were responsible for the obtaining of other compounds, such as villanovane II and III (**132**–**133**) and the diterpenoids with atysane skeleton, 16*α*,17-dihydroxy-9(11)-ent-atis-19-oic acid (**134**), and with a spyroring in C ring, cafespirone acid A (**135**) [71,72]. Recently, Al-Romaima also isolated compound villanovane II from the cherry pulp of *C. arabica* [76].

Some coffee *ent*-kaurane diterpenoids showed the loss of one or two methyl groups (C-18 and C-19) linked to C-4 as a degradation type and the presence of a keto group at C-3. Al-Romaima et al. isolated from the pulp of *C. arabica* two new compounds with this chemical structure, named (16*R*)-*ent*-16,17-dihydroxy,18-hydroxymethyl,19-*nor*-kaur-4-en-3-one (**139**) and (16*R*)-*ent*-16,17-dihydroxy,18-hydroxymethyl,19-*nor*-kaur-1,4-dien-3-one (**140**) [76]. Wang et al. described for the first time the compound toscarolide I (**141**), an aglycone with this skeleton linked to the palmitic acid moiety at C-17, from roasted Yunnan coffee [75]. Tricalysione A (**137**) and dehydrotricalysione A (**138**) have also been reported for similar coffee [66,71].

Finally, in 2022, an interesting study was performed by Hu et al. with a similar roasted Yunnan coffee [73]. The authors discovered the presence of different diterpenoids, such as “aromatic-type” and “*seco*-type”. For the first subclass, only one compound was isolated and named arabiol I (**136**), with an aromatic ring fused to the unsaturated A ring (∆^1,2^, similar to kahweol) and 6/6/6/6/5 ring system. The “seco-type” subclass consists of five new *ent*-kaurane diterpenoids. Decokahweol II and III (**142**–**143**) have a 10/6/5 ring system, probably formed through the cleavage of the C-5/C-10 bond. Flosecokahweols I, II, and III (**144**–**146**) showed an aromatic A ring fused to a furan ring, while the cleavage of the C-9/C-10 bond causes the rupture of the B ring. These compounds remained in the presence of a C ring and an endocyclic D ring. This is different from any coffee diterpenoid isolated over the last few decades, demonstrating the greater chemical diversity of *Coffea* spp.

## 6. Current Considerations on Biological Activities of Cafestol and Kahweol and Related *Ent*-Kaurane Diterpenoids

Despite the chemical diversity in the group of *ent*-kauranes in the *Coffea* genus, cafestol and kahweol remain the main diterpenes reported in the context of biological activities. These compounds are still cited in studies of new molecules as comparison factors. This aspect is especially observed for cafestol, whose name was attributed to a subgroup of furanic *ent*-kauranes, cafestol-type diterpenes [73,78,79].

The bioactive profile of C and K was initially marked by a negative aspect associated with hypercholesterolemia (mainly cafestol), which was comprehensively discussed in a series of studies [80,81,82,83]. This negative aspect was not a limitation, as several biological activities, such as anti-inflammatory, antitumoral, antioxidant, and hepatoprotective activities, were subsequently reported [84,85,86,87,88]. Both diterpenes showed great potential in the antitumor context, mainly as antineoplastic sensitizers, a thematic focus of review articles that consolidate studies published since 1982 [7,89,90].

The *ent*-kaurane group is known for its bioactivities, such as cytotoxic and anti-inflammatory activities (inhibition of NO production) [91,92]. Therefore, investigation of the bioactive potential of novel diterpenes often involves their association with cancer and inflammation. However, it is necessary to highlight the influence of the variable substitution pattern in the chemical skeleton on data from biological assays.

Shu et al. investigated the cytotoxic activity of twelve diterpenes isolated from roasted Arabica beans on HL-60, SMMC-7721, A-549, MCF-7, and SW480 cancer cells [66]. The five main compounds were the glycosides mascaroside I (**60**), mascaroside II (**61**), panniculoside VI (**78**), cofaryloside I (**79**), and villanovane I (**131**). None of them exhibited cytotoxic activity, including furan-type diterpenoids. For a long time, the furan ring was considered an important pharmacophoric group for the chemopreventive action of C&K [93,94]; however, the carbonyl group at C-2 appeared to interfere with the activity outcomes. Some studies are interesting for understanding how oxidation and glycosylation in the *ent*-kaurane skeleton could affect the bioactivity of these diterpenoids.

Chu et al. showed that three new mascarosides (III-V, **62-64**) isolated from Yunnan Arabica beans exhibited no antitumor activity in the same cell lines evaluated previously for mascaroside I (**60**) and II (**61**) [67]. Furthermore, Shen et al. investigated the anti-inflammatory action (inhibition of NO production in RAW.267 macrophages) of eight non-glycoside cafestol-type diterpenoids isolated from *Tricalysia fruticose* [78]. Among the inactive diterpenoids, there was a cafestol analog with a carbonyl at C-2, suggesting that oxidation in C-2 affects bioactivity, even for free diterpenoids. It is important to mention that the cytotoxic activity in A549 and HL-60 cells and the anti-inflammatory activity (inhibition of nitric oxide production) of cafestol and/or kahweol have already been reported in the literature [95,96]. It remains to be understood how proximal oxidation could compromise the reactivity of the furan ring in biological systems to the point of justifying inactivity.

## 7. Effect on Energy Metabolism

An alternative and promising direction for studying the biological activities of C&K and their structural analogs involves focusing on energy metabolism linked to antidiabetic potential. This field is interesting since the investigation of biological activities of natural products is often associated with chronic diseases, such as cancer, diabetes, and cardiovascular diseases, due to their armful clinical impact. Based on extensive research on the roles of C&K in cancer, updating the focus to diabetes and obesity is a promising goal.

The first data suggesting the potential of cafestol as an antidiabetic agent were published in 2015 [97], thirty-three years after the first study associating these diterpenes with cancer chemoprevention [50]. Mellbye et al. demonstrated in an in vitro model with INS-1 cells that cafestol (10 nM) induced an increase in insulin secretion in acute (12%) and chronic (43%) forms compared to the control, and it was also responsible for glucose uptake increase in muscle cells, with an effect similar to that of the antidiabetic drug Rosiglitazone [97]. Subsequently, in KKAY mice, an appropriate model for diabetes and obesity research, oral administration of low doses (0.4 mg/day) and high doses (1.1 mg/day) of this diterpene for ten weeks reduced fasting and non-fasting blood glucose and increased the expression of insulin receptors in the liver by 42% [98]. Other studies focusing on kahweol have demonstrated its anti-adipogenic potential. In 3T3-L1 preadipocytes, kahweol (80 µM) reduced lipid accumulation and repressed adipocyte differentiation by inhibiting the expression of PPARγ, C/EBPα, FABP4, and FASN, proteins involved in lipid storage and adipogenesis. In addition, kahweol increased glucose uptake in preadipocytes in a dose-dependent manner. Glucose tolerance tests of mice treated every two days with kahweol (100 mg·kg^−1^ by oral administration) for two weeks demonstrated that the diterpene accelerated glucose clearance. The mechanism of action is associated with the ability of diterpene to activate AMP-activated protein kinase (AMPK) [99], one of the main enzymes that coordinate cellular energy metabolism, whose signaling pathway is dysregulated in individuals with type 2 diabetes and obesity [100,101]. Due to its inducer of AMPK activity, kahweol also revealed great potential as an anti-adipogenic and insulinotropic agent.

An alternative strategy for studying the energy metabolism (homeostasis and regulation of cellular fat accumulation) of bioactive compounds involves the application of *Caenorhabditis elegans*, a nematode with genes associated with lipid metabolism that are homologous to mammalian genes [102,103]. *C. elegans* has potential use in natural product screening for different purposes, such as anti-aging, anti-infection, and anti-obesity activity, in which flavonoids are highlighted; instead, its use for diterpenes is still limited [103].

Based on the hypercholesterolemic effect of cafestol, which results from its agonist activity on FXR (Farnesoid X receptor), a nuclear receptor involved in the biotransformation of cholesterol into bile acids, as well as other metabolic pathways, Farias-Pereira et al. investigated the effects of this diterpene on the lipid metabolism of *C. elegans* [104]. Compared to the control, cafestol (60 µM) reduced fat accumulation and increased locomotor activity by 20% and 38%, respectively, after 2 days, within activities dependent on Daf-12, a nuclear factor homologous to FXR. Tub-1 and ech-1.1, the regulator proteins of energy expenditure and β-oxidation, respectively, were upregulated by cafestol, suggesting that it has multiple targets in the metabolic regulation of *C. elegans* via the Daf-12/FXR pathway.

Subsequently, the same group evaluated the activity of kahweol in nematodes [105]. Treatment with 120 µM kahweol reduced fat accumulation by 17%, resulting from the worm’s decreased food intake during the adult phase. Genes related to lipid metabolism, especially *β*-oxidation (tub-1 and ech-1) and lipolysis, were downregulated by diterpene. Due to the previously established relationship between caloric restriction and increased lifespan in *C. elegans*, Cho and Park investigated whether kahweol would impact a worm’s time of life. Kahweol (25 µM) increased the lifespan of wild-type nematodes (28%) and mutants, whose longevity does not depend on the rate of food intake [106]. Kahweol activity was shown to be dependent on daf 2 (the homolog of insulin/insulin-like growth factor-1 receptor) and aak-2 (the homolog of AMP-activated protein kinase—AMPK), highlighting the potential of this diterpene to act on distinct metabolic pathways associated with longevity, even in a preliminary manner.

In contrast to the focus on energy metabolism studies observed for C and K, only one study on coffee *ent*-kauranes diterpenes used *C. elegans*, aiming to investigate the toxicity of 2-O-β-D-glucopyranosyl-carboxyatractyligenin (**56**), the main carboxyatractyligenin described in green beans [107]. The atractylogenin derivative was lethal to the nematode (LD_50_ = 11.7 ± 1.2 mM) at a dose 20-fold higher than sulfated carboxyatractyloside, a derivative not found in coffee beans but with known toxicity reported in different models, including humans [64,65]. Despite the alarming nature of these phytotoxins, the roasting process promotes their degradation; however, the dangers of green coffee-based products cannot be neglected [65].

Wang et al. performed a screening for anti-adipogenic agents in white adipocytes (3T3-L1 cells) with thirty-five *ent*-kaurane found in coffee, including C&K and the main degradation products dehydrocafestol (**42**) and dehydrokahweol (**43**) [75]. Among the tested compounds, distributed among lactam-type, lactone-type, furan-type, and other skeleton diterpenes, only dehydrocaffarolide B—deca B (**116**) and caffarolide A (**106**), both lactone-types, decreased the lipid content of adipocytes with high potency. Compared to the control, compounds **116** and **106** were reduced to 61.6% and 65.4%, respectively. In addition to the importance of the *α*,*β*-unsaturated-*γ*-lactone moiety, observations of structure-activity relationships suggested that the hydroxyl group at C-16 is not essential for activity. However, the hydroxyl at C-3 behaves differently because substitution by methoxy or hydrogen suppresses the activity.

**Figure 9:**
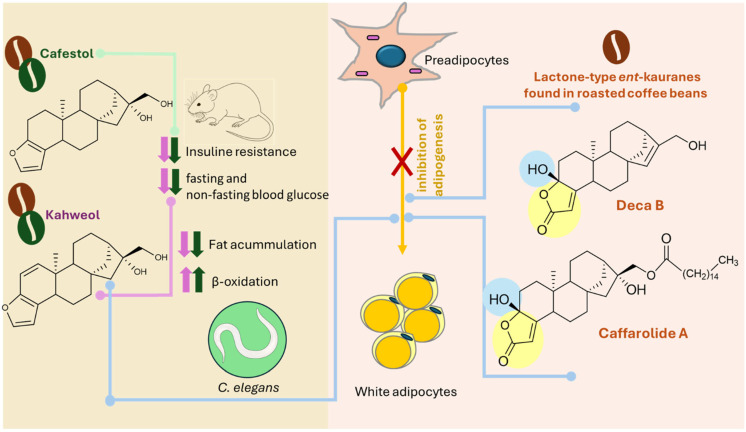
Effects of cafestol, kahweol, and lactone-type ent-kaurane diterpenoids (Deca B and Caffarolide A) found in roasted and/or green beans on energy metabolism.

Figure 9 summarizes the relationship between two classics (cafestol and kahweol) and new *ent*-kaurane diterpenoids found in roasted beans with modulations of metabolism energy considering different in vitro and in vivo models.

## 8. α-Glucosidase Inhibitory Activity of *Ent*-Kaurane Diterpenoids Associated with Molecular Docking

Among the several promising targets for treating type 2 diabetes, alpha-glucosidase has become important in natural product research [108,109]. As one of the intestinal enzymes responsible for postprandial glycemia, inhibition of alpha-glucosidase is an interesting hypoglycemic strategy. Acarbose is an approved drug that inhibits this enzyme. However, the design of new inhibitors aims to minimize adverse effects [110,111].

Alpha-glucosidase inhibition has already been reported for coffee extracts, in which polyphenols are considered the main class of compounds responsible for the hypoglycemic action [112,113]. Hu et al. were the first to investigate the potential of *ent*-kaurane diterpenes as alpha-glucosidase inhibitors, contributing to the understanding of the class role against type 2 diabetes [70]. Of the seven lactam-type *ent*-kaurane isolated from roasted beans, named cafemides A-G, five compounds exhibited activity. However, only three diterpenoids, cafemide A (**119**), E (**123**), and G (**125**), showed IC_50_ values lower than those of ursolic acid and acarbose, both positive controls. Despite the lack of deep discussion about the structure-activity relationship in this study, some notes regarding C-1 suggest that the hydroxyl group may not favor inhibitory activity. In addition, unsaturation of the acyl chain did not appear to impact the activity, since the esters of palmitic, oleic, and linolenic acid were active. Through a comparison of cafemides E and G, a double bond Δ^2,3^ may contribute to the activity instead of the epoxide ring resulting from oxidation.

Molecular docking, which predicts the binding modes and affinity of ligands for a target protein, is an in silico approach with wide applications in the screening of drugs [114]. Furthermore, docking simulations are an interesting strategy to support structure-activity relationship studies. In studies of alpha-glucosidase inhibitory activity of coffee *ent*-kauranes, the adoption of molecular docking was fundamental for a deeper understanding of the results from biological assays [71,72,74,76].

Hu et al. demonstrated that only four compounds of the fifteen diterpenes isolated from roasted beans had IC_50_ lower than the positive control (acarbose) [71]. Among the active compounds, the degradation products 15,16-dehydrocafestol (**42**) and 15,16-dehydrokahweol were observed (**43**). The Δ^15,16^ bond was considered necessary for the inhibitory activity of cafestol, whereas kahweol was inactive. By comparing the locations of the ligands in the complex, it was found that the compounds occupied the same cavity. The exception was dehydrocaffaldehyde A (**69**), the most potent diterpene (IC_50_ 23.23 ± 1.03 μM) that showed a spatial orientation opposite to the others and allowed the formation of two hydrogen bonds with PHE314 and ARG315 amino acid residues. Furthermore, unlike other active diterpenoids, dehydrocaffaldehyde A (**69**) did not form a hydrogen bond involving the substituent in C-17 (aldehyde), justifying its lowest IC_50_ among the series of active diterpenes.

In contrast to other studies that employed diterpenoids from coffee beans, Al-Romaima et al. investigated the action of thirteen diterpenes isolated from Arabica coffee pulp and showed that only caffruenol B (**128**) had a lower IC_50_ than acarbose. In addition, other diterpenoids exhibited moderate activity [76]. Docking simulations indicated that a hydrogen bond was formed between the hydroxyl group at C-17 and ARG-442 as the main interaction in the caffruenol B-protein complex. Although caffruenol B (**128**) had the lowest IC_50_ value, it did not correspond to the lowest docking energy (−8.5 kcal/mol) within the diterpene series. Since these are predictions of ligand-protein interactions, lower energies do not correspond to greater activity, as the experimental data depend on different factors [76]. In the studies previously cited, the most potent compounds were not those with the lowest docking energies [71,72]. These observations highlight that in silico approaches must always be correlated with experimental data. Even if the binding energy and potency are inconsistent, understanding the ligand poses and their interactions with amino acid residues is relevant for elucidating the structure-activity relationship.

**Figure 10:**
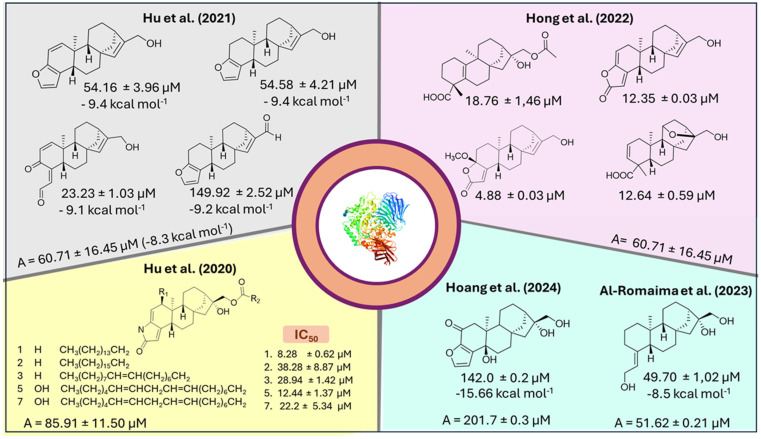
Coffee *ent*-kaurane derivatives found in roasted beans and pulp have high and moderate *α*-glucosidase inhibitory activity. All studies used acarbose (**A**) as a positive control. Binding energy (kcal mol^−1^) is indicated for those studies that compared this criterion to molecular docking simulations. [70,71,72,76,77].

The chemical structure, IC_50_ values, and docking energy of the coffee *ent*-kaurane diterpenoids with high and moderate potency for α-glucosidase inhibition are shown in Figure 10.

## 9. Another Biological Activities

Expanding the association between diterpenoids and common chronic diseases (cancer and diabetes), some studies have focused on atypical biological activities over the last few years. Wang et al. reported three lactone-diterpene esters (caffarolides) from Arabica beans with antiplatelet activity [68]. In the discussion of structure-activity relationships, hemiketal at C-3, variation of substitutions at C-1 (hydroxyl, hydrogen, and methoxy), and unsaturation profile in the acyl chain of fatty acids were considered determinants of activity variability. Among these observations, the hemiketal moiety was the main factor since cafarolides without a hydroxyl group at C-3 did not show activity, regardless of the degree of unsaturation of the acyl chain or the substitution pattern at C-1.

Hu et al. evaluated the T-type Ca^2+^ (Cav.3) channel inhibitory activity of twenty-six diterpenoids, including novel and known from roasted Arabica coffee beans [73]. They were classified as cafestol-type, kahweol-type, and coffee diterpenoid acid derivatives. Nine kahweol-types exhibited significant activity at a low concentration (10 µM). Arabiol (**136**), decokahweol II (**142**), and decokahweol III (**143**) were the most potent, with IC_50_ values of 2.9, 2.3, and 0.68 µM, respectively. These kahweol-types, described as new Cav.3 inhibitors, have demonstrated potential for epilepsy and neuropathic pain therapy, a clinical picture where these channels are important promising targets.

## 10. Perspectives of Coffee *Ent*-Kaurane Diterpenoids

The synthesis of natural product derivatives is a strategy to obtain molecules with greater therapeutic potential, considering their increased efficacy and safety. Plants remain an important source of specialized metabolites that inspire the design, discovery, and development of new drugs [115]. Kaurenoic acid and oridonin stand out for the synthesis of derivatives for different therapeutic purposes in the *ent*-kaurene group [116]. However, studies on a synthetic approach that uses coffee *ent*-kaurane as the starting molecule are scarce.

Badalamenti et al. subjected atractiligenin to different reactions (oxidation, bromination, and elimination) and obtained an *ent*-kaurane skeleton with an α,β-unsaturated system that showed antiproliferative activities similar to those of the antineoplastic drug cisplatin [117]. This study was of interest to expand the bioactive profile of derivatives of a class previously seen only as toxins.

Although bioactivity studies are essential for the development of new drugs and in natural product research, an understanding of pharmacokinetics should not be neglected. In recent years, some studies have emphasized the metabolites that could be formed by human metabolism through animal models and/or human assays. Recently, Andriolo et al. and Lang et al. proposed different metabolites through cafestol (**1**) and atractyligenin-2-*O*-*β*-D-glucoside (**56**). This information is important for future studies involving biological considerations in human organisms [118,119].

Considerations of the degradation products present in roasted coffee remain important. A recent study published by Czech et al. demonstrated the formation of roasting products through the pyrolysis of mozambioside (**52**) and provided the identification of five new compounds by extensive structural elucidation. These compounds were associated with the bitter taste impression of coffee to different degrees [120].

## 11. Conclusions

In this present review, the coffee ent-kaurane diterpenoids were described, focusing mainly on recent discoveries. A total of 146 *ent*-kaurane diterpenoids isolated from the different matrices of *Coffea* spp. were related since the first article published in 1932, including the known cafestol and kahweol. Most compounds were reported twelve years later. This number of diterpenoids demonstrates great chemical diversity, with a pattern of chemical backbones. In our review, we presented these compounds in different groups, such as furan-type, oxidation-type, rearrangement-type, lacton/lactam-type, and other-type (a few compounds with unusual backbone structures).

For these coffee diterpenoids, this review detailed their several biological activities, such as the anticancer and anti-inflammatory potential of the C and K, antidiabetic and ant-adipogenic potential, *α*-glucosidase inhibition, anti-aggregation agents for thrombosis, promising Cav.3 inhibitors for epilepsy treatment, among others. For example, some lactone/lactam-type *ent*-kaurane diterpenoids are responsible for the inhibition of alpha-glucosidase and the reduction of lipid content in adipocytes. This review revealed the potential of these new coffee *ent*-kaurane diterpenoids for new biological perspectives and the design, discovery, and development of new drugs.

## Figures and Tables

**Table 1:** Phytochemical research on ent-kaurane diterpenoids from the *Coffea* genus since the last decade.

*Coffea* spp.	Plant Part	Geographical Origin	Compounds	Reference
*Coffea* L.	Roasted and green beans	Purchased in German markets	**Oxidation-type:** 55, 56, 57, 72,73	Lang et al. (2013) [65]
*C. arabica* var. *yunnanensis*	Roasted beans	Yunnan province, People’s Republic of China	**Furan-type:** 52, 53, 60, 61 **Oxidation-type:** 55, 57, 58, 76, 77, 78, 79 **Other-type:** 131	Shu et al. (2014) [66]
*C. arabica* L.	Green beans	Yunnan province, People’s Republic of China	**Furan-type:** 53, 62, 63, 64 **Oxidation-type:** 55, 57, 58, 76 **Rearrangement-type:** 92, 93 **Other-type:** 137	Chu et al. (2016) [67]
*C. arabica* L.	Green beans	Yunnan province, People’s Republic of China	**Lactone-type:** 106, 107, 108, 109, 110, 111, 112, 113	Wang et al. (2018) [68]
*C. arabica* L.	Air-dried cherries	Yunnan province, People’s Republic of China	**Oxidation-type:** 58, 84, 85, 86, 87, 88, 90 **Lactone-type:** 100, 101, 102, 103, 104, 105 **Other-type:** 127,128	Wang et al. (2019) [69]
*C. arabica* L.	Roasted beans	Yunnan province, People’s Republic of China	**Lactam-type:** 119, 120, 121, 122, 123, 124, 125	Hu et al. (2020) [70]
*C. arabica* L.	Roasted beans	Yunnan province, People’s Republic of China	**Furan-type:** 1, 2, 42, 43, 46, 65, 66, 69 **Oxidation-type:** 74, 75 **Rearrangement-type:** 95 **Lactone-type:** 115 **Other-type:** 129, 134	Hu et al. (2021) [71]
*C. arabica* L. (cultivar S288)	Roasted beans	Yunnan province, People’s Republic of China	**Oxidation-type:** 83, 90, 91 **Rearrangement-type:** 94, 96, 97, 99 **Lactone-type:** 117, 118 **Other-type:** 132, 133, 135, 138	Hong et al. (2022) [72]
*C. arabica* L.	Roasted beans	Yunnan province, People’s Republic of China	**Rearrangement-type:** 98 **Other-type:** 136, 142, 143, 144, 145, 146	Hu et al. (2022) [73]
*C. canephora*	Trunks	Lam Dong province, Vietnam	**Oxidation-type:** 80, 81	Nguyen et al. (2023) [74]
*C. arabica* L.	Roasted beans	Yunnan province, People’s Republic of China	**Furan-type:** 71 **Lactone-type:** 114, 116 **Other-type:** 141	Wang et al. (2023) [75]
*C. arabica* L.	Cherries Pulp	Yunnan province, People’s Republic of China	**Furan-type:** 67, 68, 70 **Oxidation-type:** 85, 89 **Lactone-type/Lactam-type:** 101, 126 **Other-type:** 128, 130, 132, 139, 140	Al-Romaima et al. (2024) [76]
*C. canephora*	Trunks	Lam Dong province, Vietnam	**Furan-type:** 1, 82 **Oxidation-type:** 55 **Lactone-type:** 100	Hoang et al. (2024) [77]
